# Molecular monitoring of insecticide resistance in major disease vectors in Armenia

**DOI:** 10.1186/s13071-024-06139-2

**Published:** 2024-02-06

**Authors:** Lusine Paronyan, Lilit Babayan, Haykuhi Vardanyan, Arsen Manucharyan, Kyriaki Maria Papapostolou, Sofia Balaska, John Vontas, Konstantinos Mavridis

**Affiliations:** 1 National Center for Disease Control and Prevention, MOH, Yerevan, Republic of Armenia; 2 Institute of Molecular Biology and Biotechnology, Foundation for Research and Technology-Hellas, 70013 Heraklion, Greece; 3 Department of Crop Science, Pesticide Science Laboratory, Agricultural University of Athens, 11855 Athens, Greece

**Keywords:** Malaria, Arboviruses, Leishmaniasis, Mosquitoes, Sand flies, Molecular insecticide resistance

## Abstract

**Background:**

Armenia is considered particularly vulnerable to life-threatening vector-borne diseases (VBDs) including malaria, West Nile virus disease and leishmaniasis. However, information relevant for the control of the vectors of these diseases, such as their insecticide resistance profile, is scarce. The present study was conducted to provide the first evidence on insecticide resistance mechanisms circulating in major mosquito and sand fly populations in Armenia.

**Methods:**

Sampling sites were targeted based mainly on previous historical records of VBD occurrences in humans and vertebrate hosts. Initially, molecular species identification on the collected vector samples was performed. Subsequently, molecular diagnostic assays [polymerase chain reaction (PCR), Sanger sequencing, PCR-restriction fragment length polymorphism (RFLP), quantitative PCR (qPCR)] were performed to profile for major insecticide resistance mechanisms, i.e. target site insensitivity in voltage-gated sodium channel (*vgsc*) associated with pyrethroid resistance, acetylcholinesterase (*ace-1*) target site mutations linked to organophosphate (OP) and carbamate (CRB) resistance, chitin synthase (*chs-1*) target site mutations associated with diflubenzuron (DFB) resistance and gene amplification of carboxylesterases (CCEs) associated with resistance to the OP temephos.

**Results:**

*Anopheles* mosquitoes were principally represented by *Anopheles sacharovi*, a well-known malaria vector in Armenia, which showed no signs of resistance mechanisms. Contrarily, the knockdown resistance (*kdr*) mutations V1016G and L1014F/C in the *vgsc* gene were detected in the arboviral mosquito vectors *Aedes albopictus* and *Culex pipiens*, respectively. The *kdr* mutation L1014S was also detected in the sand fly, vectors of leishmaniasis, *Phlebotomus papatasi* and *P. tobbi*, whereas no mutations were found in the remaining collected sand fly species, *P. sergenti*, *P. perfiliewi* and *P. caucasicus*.

**Conclusions:**

This is the first study to report on molecular mechanisms of insecticide resistance circulating in major mosquito and sand fly disease vectors in Armenia and highlights the need for the establishment of systematic resistance monitoring practices for the implementation of evidence-based control applications.

**Graphical Abstract:**

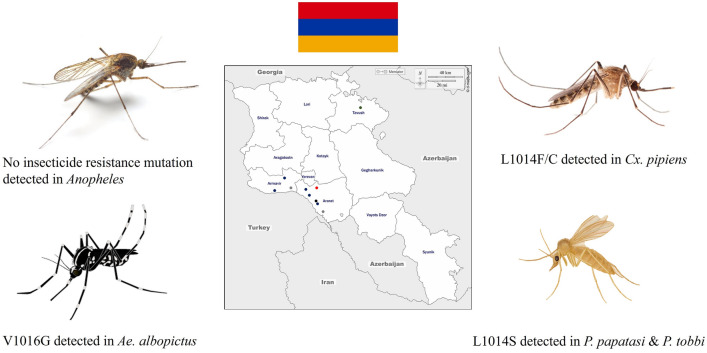

**Supplementary Information:**

The online version contains supplementary material available at 10.1186/s13071-024-06139-2.

## Background

The geographical and climatic conditions of Armenia, a landlocked country in the Caucasus region of Eurasia, are favourable for arthropod vectors. Ninety-five percent (95.0%) of Armenia’s territory is considered susceptible to especially dangerous vector-borne diseases (VBDs) [1, 2].

Historically, a number of VBDs have been prevalent in Armenia, such as malaria [3], arboviral diseases [4–6] and leishmaniasis [7, 8]. Although a malaria elimination process was completed in 2005 in Armenia, the re-emergence of autochthonous malaria remains a constant threat [3]. This is also true for arboviral diseases; a previously conducted large entomological survey identified 125 distinct strains of 10 arboviruses, including major public health threats, such as West Nile virus (WNV) [2, 4, 5]. Leishmaniasis is another significant public health problem in Armenia, where both visceral (VL) and cutaneous (CL) forms have been recorded; from 1999 to 2021, 202 indigenous VL cases were registered, with 7 of those being fatal [1, 7, 8].

Mosquitoes of the genera *Anopheles*, *Aedes* and *Culex* are some of the most important vectors of infectious diseases in Armenia, relating to public health crises such as malaria, WNV and dengue. The vectors are spread over the territory of Armenia for most of the year (from April to November). Indeed, a comprehensive study performed in 2016 identified a total of 29 different species of mosquitoes. It identified 6 anophelines (including major malaria vectors *Anopheles sacharovi* and *An. maculipennis* s.s.), 10 *Aedes* (including *Aedes albopictus*, which can transmit dengue, chikungunya and other arboviruses) and 8 *Culex* species [including *Culex pipiens*, the major vector of WNV [2]. Regarding *Phlebotomus* sand flies, entomological surveys have shown an increase in their populations [1], referring also to key vectors such as *Phlebotomus papatasi* and *P. sergenti* [9]. Such a wide variety of vectors can complicate local transmission of endemic communicable diseases by causing the re-emergence of previously eliminated VBDs and is an important factor in the introduction of newly detectable diseases [10].

The use of chemical insecticides remains a fundamental and highly effective intervention tool for reducing all these vector populations, thus controlling the spread of VBDs during outbreaks [11]. In Armenia, in particular, the pyrethroids cypermethrin and cyfluthrin are used to control vectors in leishmaniasis and malaria foci, respectively [1]. However, the development of insecticide resistance in disease vectors threatens the effectiveness of vector control strategies [12–14]. In Armenia, resistance to pyrethroids has already been documented by Paronyan et al. in *An. maculipennis* s.l. and *Cx. pipiens* mosquitoes at the phenotypic level using WHO adult susceptibility bioassays [15].

Investigation of the molecular mechanisms underlying insecticide resistance in wild vector populations through regular monitoring of resistance markers could lay the foundation for decision-making processes in vector control campaigns within the framework of evidence-based Insecticide Resistance Management (IRM) [15, 16].

Mechanisms of insecticide resistance include target-site resistance due to mutations in the target where the insecticide attacks and metabolic resistance due to overexpression of detoxification genes such as cytochrome P450s (CYPs), glutathione S-transferases (GSTs) and carboxylesterases (CCEs) [13]. Regarding target site insensitivity, knockdown resistance (*kdr*) mutations in the  voltage-gated sodium channel (*vgsc*) gene have been previously associated with pyrethroid resistance in *Anopheles* (mutations: L1014F/C/S) [17], *Culex* (mutations: L1014F/C/S) [18] and *Aedes* (mutations: F1534C/L/S; V1016G/I; I1532T) [19] mosquito vectors as well as in *Phlebotomus* sand fly vectors (mutations L1014F/S) [14]. Resistance to acetylcholinesterase inhibitors organophosphates (OPs) and carbamate (CRB) is mediated by *Ace-1* target-site mutations (G119S) and is found in *Anopheles* spp. and *Cx. pipiens* mosquitoes [20]. Chitin synthase (*chs-1*) mutations (I1043L/M/F), recorded so far in *Cx. pipiens* populations, have been strongly linked with resistance to the larvicide diflubenzuron (DFB) [21]. Concerning metabolic resistance, the overexpression of *CCEs CCEae3a* and *CCEae6a* is associated with temephos resistance in *Ae. albopictus* [22]. These mechanisms have already been reported mostly in mosquito (*kdr*, *ace-1*, *chs-1* mutations, *CCE* upregulation) [15, 23–26] and sand fly [14] populations (*kdr* mutations) in Europe and neighbouring countries; however, relevant data are lacking for Armenia.

We performed the present study to provide a first snapshot of the molecular insecticide resistance mechanisms operating in major disease vectors in Armenia (*Anopheles*, *Aedes*, *Culex* mosquitoes and *Phlebotomus* sand flies) and possibly detect emerging resistance traits for better monitoring and management of resistance as early as possible.

## Methods

### Collection of mosquitoes and sand flies and sample handling

**Fig. 1:**
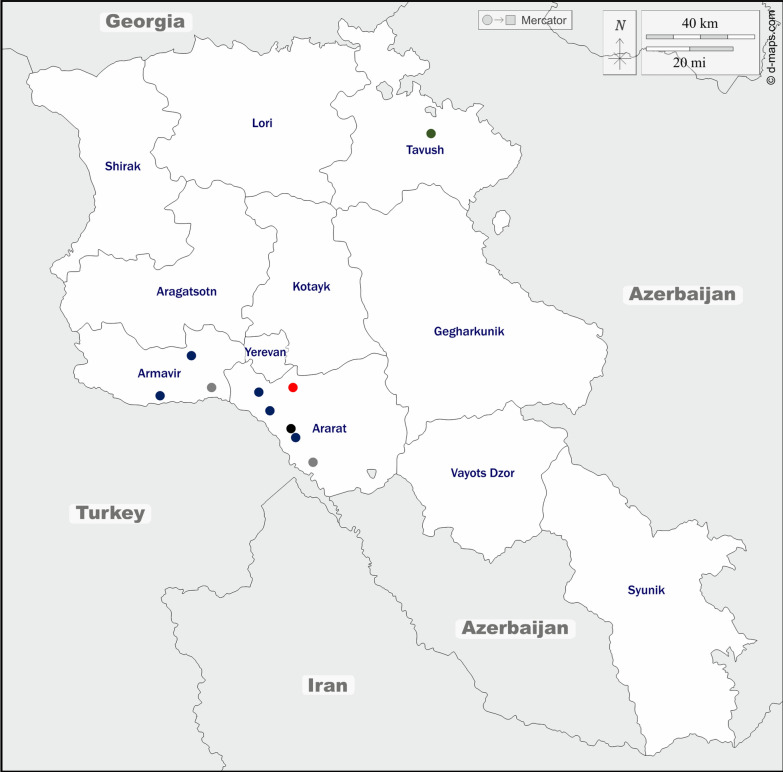
Mosquito (*Anopheles*: blue dots; *Culex*: black dot; both *Anopheles* and *Culex*: grey dots; *Aedes*: green dot) and sand fly (red dot) collection sites of the study. The base layers of the left panel’s maps were obtained from d-maps.com [59]

Samples were collected between September and October 2021 in the Ararat (including Yerevan), Armavir, Tavush and Yerevan provinces at the sites depicted in Fig. 1 and described in detail in Additional file 1: Table S1.

Ararat Province of Armenia is an area of 2090 km^2^, located at the southeast of the Ararat Valley. The annual amplitude of the average monthly air temperature in the Ararat Plain is the highest in the entire South Caucasus and reaches > 31 ℃. On average, about 220 mm of precipitation falls per year. The Ararat region comprises mostly marshy areas. The total area of ​​the city of Yerevan is 223 km^2^. Yerevan is in the northeastern part of the Ararat valley, at an altitude of 900–1200 m above sea level. Yerevan, historically and currently, is one of the visceral leishmaniasis hotspots in Armenia. Naturally, about 70 animal species may act as reservoirs for *Leishmania* parasites, including humans [1].

The Armavir region of Armenia is in the southwest of the country, in the Ararat valley. Armavir has an area of 1242 km^2^. The province is entirely located at the heart of the Ararat plain, mainly consisting of agricultural lands, with an average height of 850 m above sea level. The climate of the Armavir region is strictly continental. Throughout the year, temperatures usually range from – 7 ℃ to 34 ℃. Annual precipitation is about 305 mm. The epidemic susceptibility in terms of VBDs in the region is like in Ararat. In 2021, one case of quartan malaria was recorded in the Echmiadzin region of Armavir in a patient with no travel history [1].

Tavush is a province of Armenia located in the northeast, bordered by Georgia from the north and Azerbaijan from the east. Tavush has an area of 2704 km^2^. More than 50.0% of Armenia's forest resources are in the Tavush region. The average annual temperature is about 10 ℃. Precipitation falls up to 563 mm per year. The main breeding sites for mosquitoes are swamps that form along river floodplains. Because of the frequent movement of riverbeds, backwaters are formed in the valley, as well as many small reservoirs, which are the main breeding grounds for mosquitoes. The close connection between mosquito breeding and river valleys is especially noticeable here [1].

Mosquitoes and sand flies were collected using Center for Disease Control (CDC)-type light traps supplied with 1.5 kg CO_2_. All insect collections were immediately stored in 70.0% ethanol and transferred to the laboratory where they were stored at − 20 ℃. Mosquitoes were morphologically identified to the genus level [27] and molecularly to the species level, and sand flies were molecularly identified at the species level. The sampling sites were selected based on a combination of factors prioritized per: (i) historical records of VBD occurrences in humans (e.g. Ararat, Armavir: high risk, former malaria hyperendemic zone, Yerevan: hotspot of visceral leishmaniasis) and vertebrate hosts, (ii) previous knowledge on sites with increased mosquito and sand fly populations and (iii) insecticide applications in leishmaniasis foci and intense use of pesticides for agricultural purposes (including the pyrethroids lambda-cyhalothrin and deltamethrin).

### DNA extraction from mosquitoes and sand flies

Genomic DNA was extracted from a total of *N* = 104 individual insects (74 mosquitoes and 30 sand flies, Additional file 1: Table S1), using the DNAzol protocol according to the manufacturer’s instructions (Invitrogen, Carlsbad, CA, USA), with the following modifications: 200 μl DNAzol reagent for homogenization, 100 μl absolute ethanol for DNA precipitation, air drying for 15 min on a heat-block set at 50 ℃ and solubilization of DNA in 15 μl DNase/RNase-free water. Samples were stored at − 20 ℃ until PCR analyses.

### Molecular identification of mosquito and sand fly species

Mosquito species were molecularly identified as previously described [28, 29]. Briefly, members of *Cx. pipiens* were distinguished based on polymorphisms in the intron region of the acetylcholinesterase-2 gene (ace-2) and use of species-specific primers; samples that were identified as *Cx. pipiens* were further analysed to biotype (relying on polymorphisms in the 5ʹ flanking region of the microsatellite locus CQ11 [30]. *Aedes albopictus* identification was confirmed using the internal transcribed spacer two gene (ITS2) as described in [31]. *Anopheles* mosquitoes were molecularly differentiated by partial amplification of the ITS2 and mitochondrial cytochrome c oxidase I gene (COI) [32, 33]. Sand fly species discrimination was performed based on the COI genomic fragment, as previously described [29, 34].

### Monitoring of molecular mechanisms of insecticide resistance

Using the outer primers and PCR conditions provided in [18], each *Culex* mosquito was genotyped for the presence of the *kdr* mutations L1014F/C/S in the *vgsc* gene. *Anopheles* specimens were also genotyped for the *kdr* mutations L1014F/C/S following the protocol described in [35]. *Anopheles* and *Culex* mosquitoes were tested for insensitive acetylcholinesterase (*ace-1*) mutations using the PCR-RFLP method as described in [20]. Monitoring of the *kdr* mutations V1016G (*vgsc* domain II) and I1532T and F1534C/L/S (*vgsc* domain III) in *Ae. albopictus* was performed using PCR amplification and sequencing of the relevant domain areas enclosing the mutation sites, as detailed in [36]. The presence of carboxylesterases 3 (*CCEae3a)* and 6 (*CCEae6a*) gene amplification events in *Ae. albopictus* were assayed as detailed in [24]. *Aedes albopictus* and *Cx. pipiens* specimens were also screened for the presence of the mutations I1043L/M/F in the *chs-1* gene, as described in [24] and [21], respectively.

The presence of *kdr* mutations L1014F/S in individual sand fly samples was monitored by genotyping the *vgsc* domain IIS6, using the following de novo designed primers for PCR amplification: forward: 5ʹ-TTCCCAGACGGTGAAATGCC-3ʹ; reverse: 5ʹ-TCATTGTCTGCAGTTGGTGCT-3ʹ. The PCR reaction was performed using the KAPA Taq PCR Kit (KAPA Biosystems, Wilmington, MA, US) using 1.0 ul genomic DNA as a template with the following thermal protocol: 95 ℃ for 5 min, 35 cycles (95 ℃ for 30 s, 58 ℃ for 40 s, 72 ℃ for 30 s) and 72 ℃ for 5 min. The generated PCR fragments of approximately 250 bp were purified after visualization in agarose gel (1.5% *w*/*v*) using the Nucleospin PCR & Gel Clean-Up Kit (Macherey Nagel, Dueren, Germany) and then subjected to Sanger sequencing (GENEWIZ, Azenta Life Sciences, Germany) using the forward primer. Sequences were analysed using the BioEdit sequence alignment editor 7.2 (https://bioedit.software.informer.com/7.2/). We additionally developed an assay to determine the possible presence of the N1575Y super *kdr* (*skdr*) mutation, associated with enhanced pyrethroid resistance in mosquito vector species, based on the published sequences of *Anopheles gambiae* (XM_318122, NCBI) and *Phlebotomus papatasi* (PPAI003017-RA, VectorBase). Direct sequencing (Sanger) of the *vgsc* gene was applied to all samples carrying the conventional *kdr* mutations L1014F/S to detect any presence of the *skdr* mutation. PCR reactions (25 ul) were performed, containing 1 × PCR Buffer A (Kapa Biosystems), 2 mM MgCl2, 0.30 mM each dNTP, 0.3 μM of each primer (Fskdr: 5-CACGCTCAACCTGTTCATTGG-3ʹ and Rskdr: 5ʹ-AGGAAGTCCAGCGTCTTGC-3ʹ), 1.25U Kapa Taq DNA Polymerase (Kapa Biosystems) and 1.0 ul genomic DNA. PCR was performed using the following temperature cycling conditions: 5 min at 95 ℃, followed by 30 s at 95 ℃, 40 s at 56 ℃, 30 s at 72 ℃ for 35 cycles and 5 min at 72 ℃ for the final extension. PCR products (312 bp) were then separated by agarose gel (1.5% *w*/*v*) electrophoresis, cleaned up using the Nucleospin PCR & Gel Clean-Up Kit (Macherey Nagel, Dueren, Germany) and sequenced from both directions by GENEWIZ (Azenta Life Sciences, Germany).

## Results

### Mosquito and sand fly species identification

Most *Anopheles* mosquitoes of Ararat Province (93.8%) belonged to the *An. sacharovi* species, with a few individuals identified as *An. claviger* and *An. hyrcanus*. In Armavir Province, all *Anopheles* mosquitoes were identified as *An. maculipennis* s.s. (Table 1).

**Table 1:** Mosquito and sand fly species composition in the study areas

Vector	Population	Species ID/biotype	*N* (%)
*Anopheles* mosquitoes	Ararat (Jrarat, Hovtashen, Masis, Taperakan, Ararat)	*An. sacharovi*	30 (93.8%)
		*An. claviger*	1 (3.1%)
		*An. hyrcanus*	1 (3.1%)
	Armavir (Metsamor, Janfida)	*An. maculipennis* s.s	3 (100.0%)
*Aedes* mosquitoes	Tavush (Ijevan)	*Ae. albopictus*	26 (100.0%)
*Culex* mosquitoes	Armavir (Jrarat)	*Cx. pipiens* biotype* pipiens*	4 (100.0%)
	Ararat (Shahumyan, Ararat)	*Cx. pipiens* biotype* pipiens*	8 (88.9%)
		*Cx. pipiens* biotype* molestus*	1 (11.1%)
*Phlebotomus* sand flies	Yerevan (Jrashen)	*P. papatasi*	19 (65.5%)
		*P. tobbi*	5 (17.2%)
		*P. sergenti*	3 (10.3%)
		*P. perfiliewi*	1 (3.5%)
		*P. caucasicus*	1 (3.5%)

Regarding *Culex* mosquitoes, the Armavir region sampling site consisted only of the *Cx. pipiens* biotype *pipiens*, whereas both *Cx. pipiens* biotype *pipiens* (88.9%) and *Cx. pipiens* biotype *molestus* (11.1%) were detected in Ararat.

All *Aedes* mosquitoes sampled in Tavush Province were *Ae*. *albopictus* species. *Aedes albopictus* was the only species of the *Aedes* genus mosquitoes sampled in the Tavush region (Table 1).

Concerning the sand fly species composition in Yerevan Province, *P. papatasi* was the dominant species (65.5%), followed by *P. tobbi* (17.2%) and *P. sergenti* (10.3%), whereas *P. perfiliewi* and *P. caucasicus* were detected at 3.5% each (Table 1). Representative electropherograms of the COI gene region for each of the sand fly species detected are presented in Additional file 1: Figure S1.

### Monitoring of insecticide resistance mechanisms in mosquito and sand fly vectors

*Anopheles* mosquitoes from all study sites were analysed for the presence of *kdr* mutations L1014F, L1014C, and L1014S, associated with pyrethroid resistance and of the G119S mutation associated with OPs and CRB resistance, but none of these mutations were detected.

*Aedes albopictus* mosquitoes were tested for a total of six *kdr* mutations (i.e. V1010G, V1010GI, I1532T, F1534C, F1534L and F1534S). The V1016G mutation was detected, albeit at a very low frequency (mutant allelic frequency = 1.9%; 1 heterozygote mosquito). The presence of *CCEae3a* and *CCEae6a* amplification, associated with OP (temephos) resistance, as well as the presence of *chs-1* mutations (I1043L, I1043M, and I1043F), linked with diflubenzuron (DFB) resistance, were also investigated in the study’s *Ae. albopictus* mosquitoes, but neither of these two mechanisms was detected.

*Culex pipiens* mosquitoes were analysed for the presence of *kdr* mutations L1014F, L1014C and L1014S. Of those mutations, L1014F was detected with a mutant allelic frequency of 25.0% in the Armavir population and of 14.3% in the Ararat population, which additionally harboured the L1014C mutation at a frequency of 28.6%. None of the OP/CRB and DFB resistance mechanisms tested (G119S and I1043L/M/F mutations, respectively) were detected (Table 2).

**Table 2:** Investigation of insecticide resistance mechanisms in the study’s *Culex pipiens* populations

Population	Associated insecticides				
	Pyrethroids			OP/CRB	DFB
	Resistant mutation allelic frequencies (hetero/homo), *V* of alleles				
	L1014F	L1014C	L1014S	G119S	I1043L/M/F
Armavir (Jrarat)	25.0%	0.0%	0.0%	0.0%	0.0%
	(2/0)	(0/0)	(0/0)	(0/0)	(0/0)
	N = 8	N = 8	N = 8	N = 8	N = 8
Ararat (Ararat, Shahumyan)	14.3%	28.6%	0.0%	0.0%	0.0%
	(0/2)	(1/2)	(0/0)	(0/0)	(0/0)
	N = 18	N = 18	N = 18	N = 8	N = 8

OP/CRB: organophosphates/carbamates; DFB: diflubenzuron

The collected sand fly population from Yerevan (Jrashen) was analysed for the presence of *kdr* mutations L1014F and L1014S, and the latter was found in *P. papatasi* at a mutant allelic frequency of 42.1% and in *P. tobbi* at a frequency of 10.0% (Table 3 and electropherograms of wild-type and mutant samples in Additional file 1: Figure S2). *Kdr*-positive samples were additionally screened for the presence of the N1575Y *skdr* mutation, which, however, was not detected (Table 3 and Additional file 1: Figure S3). No *kdr* mutations were detected in the remaining sand fly species (*P. sergenti*, *P. perfiliewi* and *P. caucasicus*).

**Table 3:** Investigation of pyrethroid resistance mechanisms in the study’s sand fly population

Species	Resistant mutation allelic frequencies (hetero/homo), *N* of alleles		
	*Kdr* L1014F	*Kdr* L1014S	Super *kdr* N1575Y*
*Phlebotomus papatasi*	0.0%	42.1%	0.0%
	(0/0)	(4/6)	(0/0)
	*N* = 38	*N* = 38	*N* = 16
*Phlebotomus tobbi*	0.0%	10.0%	0.0%
	(0/0)	(1/0)	(0/0)
	*N* = 10	*N* = 10	*N* = 2
*Phlebotomus sergenti*	0.0%	0.0%	N/A
	(0/0)	(0/0)	
	*N* = 6	*N* = 6	
*Phlebotomus perfiliewi*	0.0%	0.0%	N/A
	(0/0)	(0/0)	
	*N* = 2	*N* = 2	
*Phlebotomus caucasicus*	0.0%	0.0%	N/A
	(0/0)	(0/0)	
	*N* = 2	*N* = 2	

N/A: not analysed; *tested only in samples with a positive *kdr* result

## Discussion

Armenia faces a considerable risk of VBDs [1] like malaria [3], WNV disease [4, 6] and leishmaniasis [7, 8]. Unfortunately, essential information supportive of the control of corresponding disease vectors, including their resistance to insecticides, is rather limited. To address this gap, our study aimed to present initial findings on the prevalence of insecticide resistance mechanisms among the primary mosquito and sand fly vectors in Armenia.

The presence of *An. sacharovi*, the historically known vector of malaria in Armenia [37], was confirmed, in line with previously reported data. We investigated all known resistance mechanisms for this vector, i.e. *kdr* mutations associated with pyrethroid and DTT resistance and the G119S mutation associated with OP and CRB resistance, but none were detected. No data are available from previous studies in Armenia on the resistance status of *An. sacharovi.* Such information is limited in *An. maculipennis* s.l., belonging to the same species group as *An. sacharovi* (maculipennis group), and show evidence of phenotypic pyrethroid (alphacypermethrin and cyfluthrin) resistance [15]. Data from neighbouring countries show that resistance is established in *Anopheles* populations from Turkey [38, 39] and Azerbaijan [40] (including *An. sacharovi* and *An. maculipennis* s.l.) as well as in Iran [40, 41].

Both major European arboviral vectors (*Cx. pipiens* and *Ae. albopictus*) were detected. The presence of *Ae. albopictus* was confirmed after the first report of its introduction in Armenia [2]. *Aedes albopictus* is of epidemiological concern since it is a competent vector of more than 22 arboviruses, including CHIKV and DENV, and has been implicated in major arboviral outbreaks in Europe [42, 43]. *Aedes albopictus* mosquitoes were tested for a total of six *kdr* mutations linked to pyrethroid resistance (V1010G, V1010GI, I1532T, F1534C, F1534L, F1534S), and the V1016G mutation was detected, albeit at a very low frequency (1.9%; 1 heterozygote mosquito). Although no previous data are available in Armenia, the presence of V1016G mutation has been previously recorded in neighbouring countries, at a frequency of 1.0% in Georgia and 1.9% in Turkey, and this is concerning for neighbouring countries like Iran [44], where this vector is present, but also for Europe in general [45]. The occurrence of V1016G, despite its low frequency, is alarming regarding future further resistance spread. Indeed, V1016G has been shown to confer the highest levels of resistance among *Ae. albopictus* *kdr* mutations to different pyrethroids [46]. Neither *chs-1* mutations (I1043L, I1043M, I1043F), linked with DFB resistance, nor *CCEae3a* and *CCEae6a* amplification events, associated with OP (tempephos) resistance, were detected in any of the specimens analysed.

Regarding *Cx. pipiens*, both *Cx. pipiens* s.s. and the anthropophilic *Cx. pipiens* biotype *molestus* were detected, in line with previous findings [2]. *Culex pipiens* represents the major vector for WNV, a disease with high potential to cause major outbreaks throughout Europe [47], including Armenia. The profiling of *kdr* mutations in *Cx. pipiens* mosquitoes revealed the presence of L1014F (frequency = 25.0%) in Armavir and the presence of both L1014F and L1014C (frequencies of 14.3% and 28.6%, respectively) in Ararat. This is in line with previous data showcasing the phenotypic resistance of *Cx. pipiens* populations from Armenia to the pyrethroid alpha-cypermethrin [15]. None of the known OP/CRB and DFB target site resistance mutations tested (G119S and I1043L/M/F, respectively) were detected in the *Cx. pipiens* mosquitoes that were analysed. Previously reported pyrethroid resistance records in *Cx. pipiens* populations in the broader region include those from Turkey [48, 49] (including the presence of the L1014C mutation [50]), Azerbaijan [40] and Iran [40]. Interestingly, diflubenzuron resistance has also been detected in *Cx. pipiens* from Turkey (*chs-1* mutations I1043M and I1043L) [51].

Concerning *Phlebotomus* sand flies, the primary presence of *P. papatasi*, followed by *P. tobbi*, *P. sergenti*, *P. perfiliewi* and *P. caucasicus*, is shown, a finding consistent with previous studies [9]. Most of these species are confirmed vectors of *Leishmania* parasites [52]. Yerevan, which was the sand fly sampling area of our study, is considered a hotspot of visceral leishmaniasis, accounting for > 80.0% of cases [1]. Importantly, the *kdr* mutation L1014S, associated with reduced sensitivity against pyrethroids, was detected in *P. papatasi* and *P. tobbi* at frequencies of 42.1% and 10.0%, respectively. This is the first detection of resistance at the molecular level in sand flies from Armenia to our knowledge. Data from neighbouring Turkey have previously shown the occurrence of *kdr* mutations in *P. papatasi* at a close to 50.0% allelic frequency [53].﻿ Additional studies that would also include phenotypic analyses of resistance, which seems to be established in adjacent countries like Iran [54–56] and Turkey [57], are needed to systematically monitor this phenomenon and inform vector control strategies in Armenia accordingly.

The occurrence of resistance traits that were revealed here could be due to the use of pyrethroid insecticides (cypermethrin), which are implemented in vector control programmes in Armenia [1], but it could also be related to the intense use of pesticides for agricultural purposes (including the pyrethroids lambda-cyhalothrin and deltamethrin), which has been documented in rural areas of the country [58].

## Conclusion

In conclusion, molecular profiling of known vector resistance mechanisms in Armenia led to the first detection of *kdr* mutations associated with pyrethroid resistance in major arboviral (*Ae. albopictus* and *Cx. pipiens* mosquitoes) and leishmaniasis vectors (*P. papatasi* and *P. tobbi* sand flies) to our knowledge. The limitations of this study include the limited number of specimens per species analysed and the lack of phenotypic resistance information from the tested samples. Continuous vector resistance monitoring, a principal factor in the implementation of appropriate evidence-based control programmes, should be prioritized.

### Supplementary Information

**Additional file 1: Table S1.** Detailed characteristics of the study’s sampling. **Figure S1.** Electropherograms of the COI gene region of *Phlebotomus papatasi* (**A**), *P. tobbi* (**B**), *P. sergenti* (**C**) and *P. perfiliewi* (**D**) samples. **Figure S2.** Electropherogram of a wild-type (part A) and a mutant (part B) *Phlebotomus papatasi* sand fly for the *kdr* L1014S mutation. **Figure S3.** Electropherogram of a wild-type *Phlebotomus papatasi* sample for the super *kdr* N1575Y mutation sequenced with the forward (part A) and reverse (part B) primer. **Figure S4.** Electropherogram of a wild-type (part A) and heterozygous (part B) *Aedes albopictus* sample for the *kdr* V1016G mutation sequenced with the reverse primer. **Figure S5.** Electropherogram of a wild-type *Aedes albopictus* sample for the *kdr*  I1532T and F1534L/C/S mutations sequenced with the reverse primer. **Figure S6.** Electropherogram of a wild-type *Aedes albopictus* sample for the *chs-1* I1043L/M/F mutations sequenced with the forward primer. **Figure S7.** Electropherogram of a wild-type *Anopheles sacharovi* sample for the * kdr* L1014C/F/S mutations sequenced with the forward primer. **Figure S8.** Electropherogram of a wild-type (part A: L1014) and four different mutants (part B: 1014F), (part C: 1014C) and heterozygous (part D: 1014L/F) and (part E: 1014L/C) *Culex pipiens* samples for the * kdr* L1014F/C/S mutations sequenced with the forward primer. **Figure S9.** Electropherogram of a wild-type *Culex pipiens* sample for the *chs-1* I1043L/M/F mutations sequenced with the forward primer.

## Data Availability

All data supporting the findings of this study are available within the paper and its additional information.
