# Supplementary material for: Molecular monitoring of insecticide resistance in major disease vectors in Armenia
Source: Parasit Vectors. 2024 Feb 6;17:54. doi: 10.1186/s13071-024-06139-2 (PMC10848433; doi:10.1186/s13071-024-06139-2)
Supplement: Supplementary file 1 — Additional file 1: Table S1. Detailed characteristics of the study’s sampling. Figure S1. Electropherograms of the COI gene region of Phlebotomus papatasi (A), P. tobbi (B), P. sergenti (C) and P. perfiliewi (D) samples. Figure S2. Electropherogram of a wild-type (part A) and a mutant (part B) Phlebotomus papatasi sand fly for the kdr L1014S mutation. Figure S3. Electropherogram of a wild-type Phlebotomus papatasi sample for the super kdr N1575Y mutation sequenced with the forward (part A) and reverse (part B) primer. Figure S4. Electropherogram of a wild-type (part A) and heterozygous (part B) Aedes albopictus sample for the kdr V1016G mutation sequenced with the reverse primer. Figure S5. Electropherogram of a wild-type Aedes albopictus sample for the kdr I1532T and F1534L/C/S mutations sequenced with the reverse primer. Figure S6. Electropherogram of a wild-type Aedes albopictus sample for the chs-1 I1043L/M/F mutations sequenced with the forward primer. Figure S7. Electropherogram of a wild-type Anopheles sacharovi sample for the kdr L1014C/F/S mutations sequenced with the forward primer. Figure S8. Electropherogram of a wild-type (part A: L1014) and four different mutants (part B: 1014F), (part C: 1014C) and heterozygous (part D: 1014L/F) and (part E: 1014L/C) Culex pipiens samples for the kdr L1014F/C/S mutations sequenced with the forward primer. Figure S9. Electropherogram of a wild-type Culex pipiens sample for the chs-1 I1043L/M/F mutations sequenced with the forward primer. [file 13071_2024_6139_MOESM1_ESM.docx]

**Supplementary Material**

**Supplemental Table S1** Detailed characteristics of the study’s sampling

| **Vector** | **Site (Region)** | **Site**  **(Town/Village)** | **X Coordinate** | **Y Coordinate** | **N of insects** | **Collection Date** |
| --- | --- | --- | --- | --- | --- | --- |
| *Anopheles* | Armavir | Jrarat | 40.069556 | 44.27435 | 1 | 24.09.2021 |
|  | Ararat | Hovtashen | 40.025059 | 44.454632 | 1 | 24.09.2021 |
|  | Armavir | Metsamor | 40.146058 | 44.117768 | 1 | 28.09.2021 |
|  | Armavir | Janfida | 40.04276 | 44.021487 | 2 | 27.09.2021 |
|  | Ararat | Masis | 40.063668 | 44.438699 | 9 | 24.09.2021 |
|  | Ararat | Taperakan | 39.924667 | 44.589815 | 14 | 29.09.2021 |
|  | Ararat | Ararat | 39.848148 | 44.697715 | 7 | 12.10.2021 |
| *Aedes* | Tavush | Ijevan | 40.879373 | 45.146841 | 26 | 08.10.2021 |
| *Culex* | Armavir | Jrarat | 40.069556 | 44.27435 | 4 | 24.09.2021 |
|  | Ararat | Ararat | 39.848148 | 44.697715 | 7 | 16.09.2021 |
|  | Ararat | Shahumyan | 39.938655 | 44.574312 | 2 | 24.09.2021 |
| Sand flies | Yerevan | Jrashen | 40.05325 | 44.51476 | 30 | 24.09.2021 |

Suppl. Figure S1 Electropherograms of the COI gene region of *Phlebotomus papatasi* (A), *Phlebotomus tobbi* (B), *Phlebotomus sergenti* (C) and *Phlebotomus perfiliewi* (D) samples.

Suppl. Figure S2 Electropherogram of a wild-type (part A) and a mutant (part B) *Phlebotomus papatasi* sand fly for the *kdr* L1014S mutation.


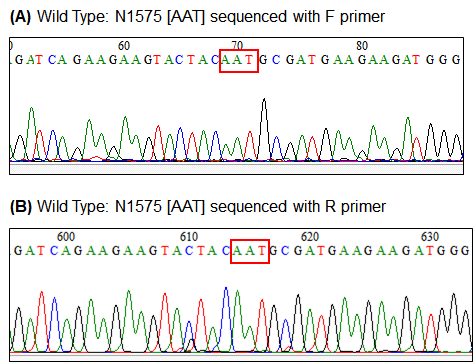


Suppl. Figure S3 Electropherogram of a wild-type *Phlebotomus papatasi* sample for the super *kdr* N1575Y mutation sequenced with the forward (part A) and the reverse (part B) primer.


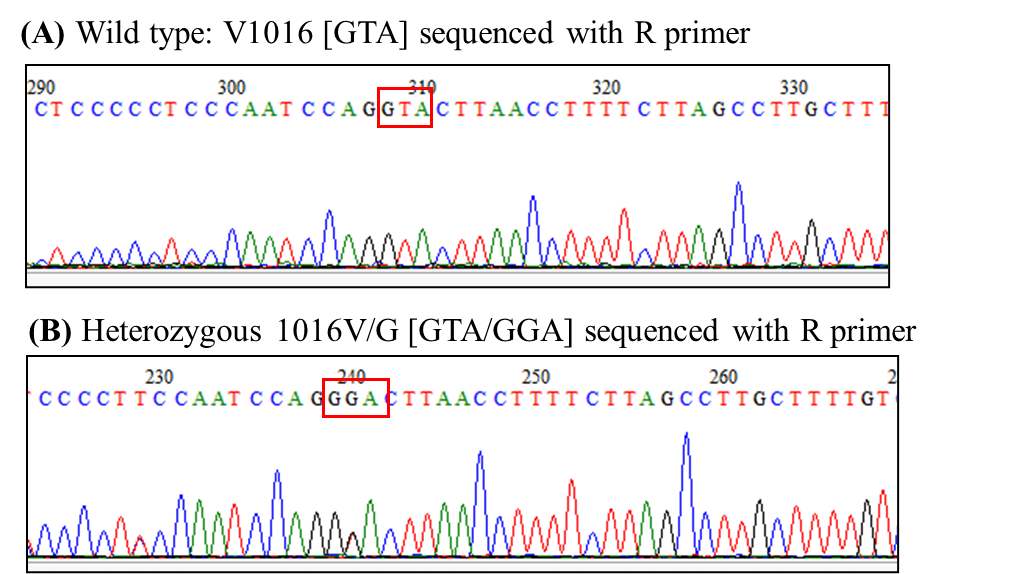


**Suppl. Figure S4** Electropherogram of a wild-type (part A) and heterozygous (part B) *Aedes albopictus* sample for the *kdr* V1016G mutation sequenced with the reverse primer.


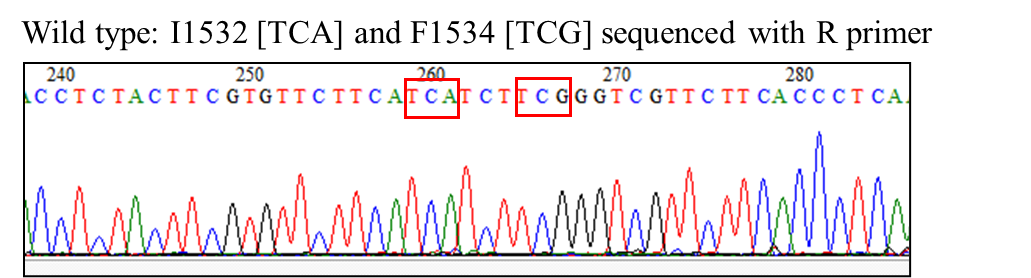


**Suppl. Figure S5** Electropherogram of a wild-type *Aedes albopictus* sample for the *kdr* I1532T and F1534L/C/S mutations sequenced with the reverse primer.


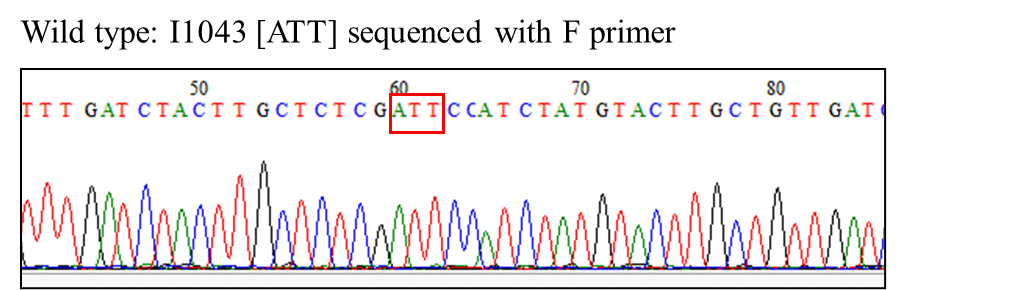


**Suppl. Figure S6** Electropherogram of a wild-type *Aedes albopictus* sample for the *chs-1* I1043L/M/F mutations sequenced with the forward primer.


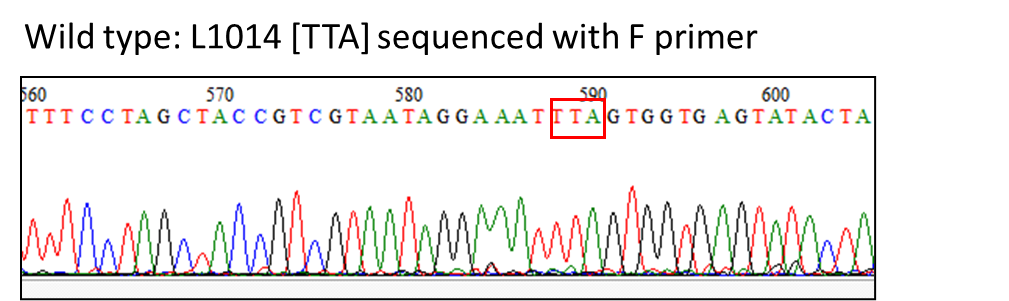


**Suppl. Figure S7** Electropherogram of a wild-type *Anopheles sacharovi* sample for the *kdr* L1014C/F/S mutations sequenced with the forward primer.


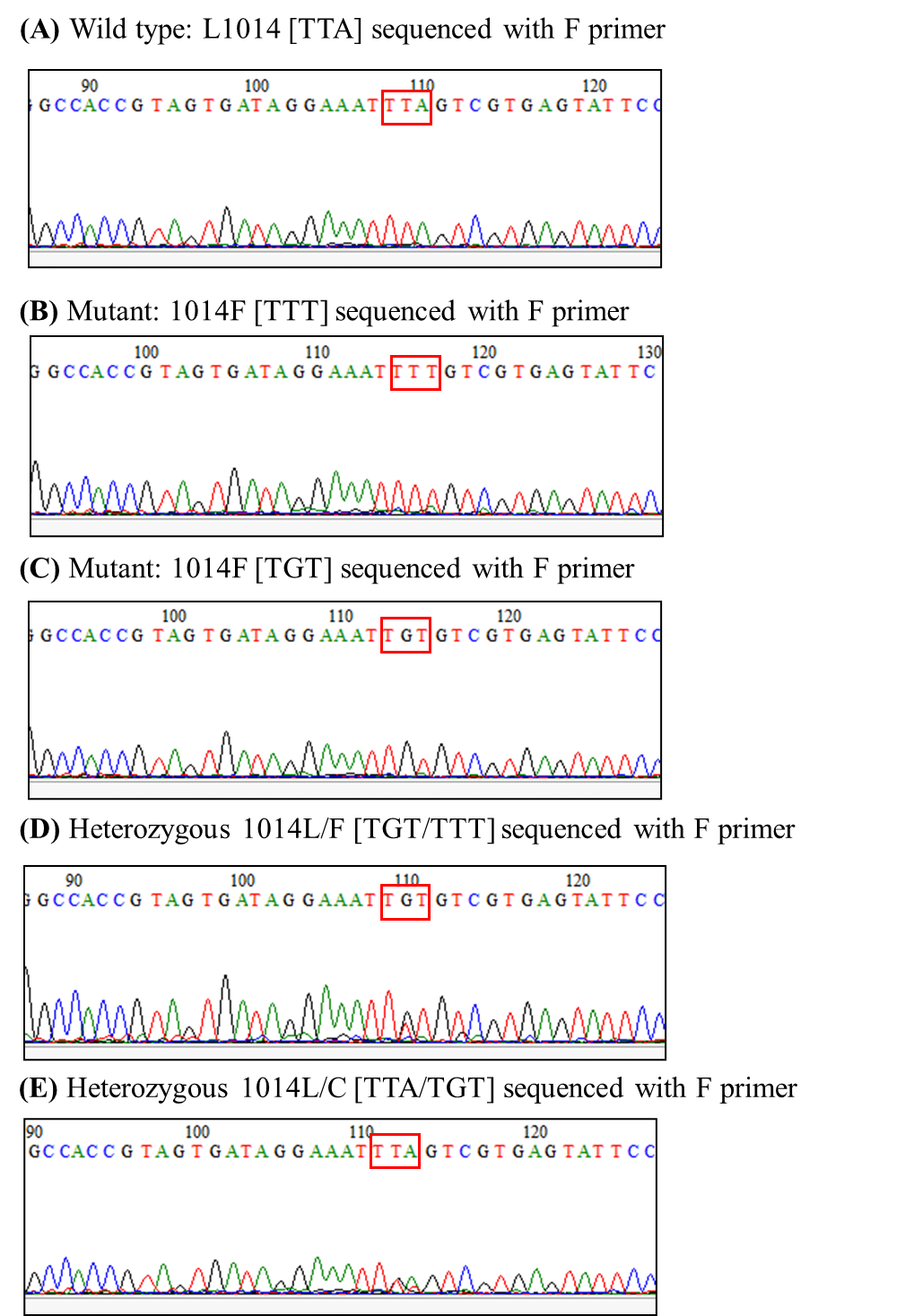


**Suppl. Figure S8** Electropherogram of a wild-type (part A: L1014) and four different mutants (part B: 1014F), (part C: 1014C), and heterozygous (part D:1014L/F) and (part E: 1014L/C) *Culex pipiens* samples for the *kdr* L1014F/C/S mutations sequenced with the forward primer.


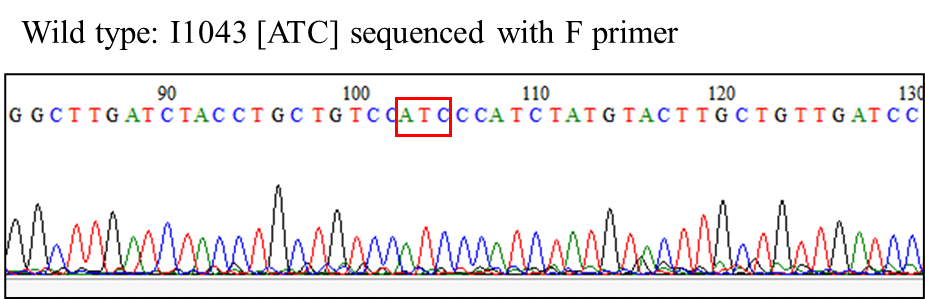


**Suppl. Figure S9** Electropherogram of a wild-type *Culex pipiens* sample for the *chs-1* I1043L/M/F mutations sequenced with the forward primer.
